# Genetic structure of *Populus* hybrid zone along the Irtysh River provides insight into plastid-nuclear incompatibility

**DOI:** 10.1038/srep28043

**Published:** 2016-06-16

**Authors:** Yan-Fei Zeng, Jian-Guo Zhang, Ai-Guo Duan, Bawerjan Abuduhamiti

**Affiliations:** 1 State Key Laboratory of Tree Genetics and Breeding, Key Laboratory of Tree Breeding and Cultivation, State Forestry Administration, Research Institute of Forestry, Chinese Academy of Forestry, Beijing, China; 2 Collaborative Innovation Center of Sustainable Forestry in Southern China, Nanjing Forestry University, Nanjing, China; 3 Forest Research Institute of the Altai Region, Xinjiang, China

## Abstract

In plants, the maintenance of species integrity despite hybridization has often been explained by the co-adaption of nuclear gene complexes. However, the interaction between plastid and nuclear sub-genomes has been underestimated. Here, we analyzed the genetic structure of a *Populus alba* and *P. tremula* hybrid zone along the Irtysh River system in the Altai region, northwest China, using both nuclear microsatellites and plastid DNA sequences. We found high interspecific differentiation, although the hybrid *P.* × *canescens* was prevalent. Bayesian inference classified most hybrids into F1, followed by a few back-crosses to *P. alba*, and fewer F2 hybrids and back-crosses to *P. tremula*, indicating a few introgressions but preference toward *P. alba*. When plastid haplotypes in parental species were distinct, *P.* × *canescens* carried the haplotypes of both parents, but showed significant linkage between intraspecific haplotype and nuclear genotypes at several microsatellite loci. Selection, rather than migration and assortative mating, might have contributed to such plastid-nuclear disequilibria. By removing later-generated hybrids carrying interspecific combinations of haplotype and nuclear genotypes, plastid-nuclear incompatibility has greatly limited the gene exchange between *P. alba* and *P. tremula via* backcrossing with hybrids, demonstrating a significant association between plastid haplotype and the proportion of nuclear admixture.

In nature, species often maintain their integrity in the absence of complete geographical or prezygotic isolation^1^,^2^,^3^,^4^. One of the explanations is that hybrids between species frequently show reduced fitness compared to their parents due to extrinsic or intrinsic factors and are thus quickly eliminated by selection^1^,^4^,^5^. Intrinsic isolation is most often attributed to the breakdown of co-adapted nuclear gene complexes (outbreeding depression)^6^,^7^,^8^. One hypothesis for such breakdown is the genetic incompatibilities described by the Bateson-Dobzhansky-Muller (BDM) model, in which substitutions that arise in different lineages cause incompatibilities when combined in a hybrid genome^9^,^10^. Sometimes, hybrids can be as fit or even fitter than their parental species, forming a new hybrid species^2^, whereas declines in fitness can be found in the second (or later) hybrid generations due to the greater severity of homozygous BDM incompatibilities^11^.

Cellular sub-genomes in the nucleus, mitochondria, and plastids communicate in complex ways and co-evolve^12^. Hybridization can lead to new interactions between the nuclear and cytoplasmic genomes as well as between different nuclear genomes, and this may result in cytonuclear incompatibilities (see references in Greiner *et al*.^13^). However, in contrast to mitochondrial-nuclear incompatibility that is of well-known evolutionary importance in animals^12^, plastid-nuclear interactions have not been extensively studied and hence the role of plastid-nuclear incompatibility in hybrid breakdown and speciation has been largely overlooked^13^,^14^, although interspecific plastid-nuclear incompatibilities have been widely reported^15^ and their implications for speciation were already recognized in the early period of formal genetics^16^. Recent studies on a few taxa have clearly shown that plastids establish hybridization barriers and play a major role in speciation^17^,^18^, but the occurrence of plastid-nuclear incompatibility in natural populations has been underestimated^12^,^13^.

Hybrid zones have often been considered as “natural laboratories for evolutionary studies”^19^, because they can contain a wide variety of genotypes that result from many generations of recombination. First, the nuclear genetic composition of individuals in a hybrid zone reflects the intensity of reproductive isolation and gene flow^20^. In general, the occurrence of multiple generations of hybrids within a hybrid zone indicates a high rate of interspecific gene flow; while a scarcity of post-F1 hybrids, such as F2 individuals and back-crosses, reveals the establishment of a barrier between the parental species^20^. In this case, the patterns of nuclear and cytoplasmic genetic structure in hybrid zones would reflect the combined effects of selection and gene flow^21^. Cytonuclear disequilibrium (CND) occurs if the cytoplasmic genomes of one species combine non-randomly with the nuclear genome of the other species due to migration, assortative mating, selection, and genetic drift^22^. In particular, cytonuclear incompatibility can contribute to the maintenance of disequilibria and influence the extent and pattern of introgression in populations^23^.

*Populus alba* L. (white poplar) and *P. tremula* L. (European aspen) are ecologically and morphologically well-differentiated species^24^,^25^,^26^. *P. alba* is widely distributed in river basins over northern Africa, southern Europe, and central Asia, while the *P. tremula* habitat is found in the mountainous regions of the boreal and temperate deciduous parts of the Eurasian continent. The distributions of these two species overlap considerably along European river systems, and can form large mosaic hybrid zones^24^,^27^. The diploid hybrid, *P.* × *canescens* (gray poplar), is frequently found close to flood-plain forests of *P. alba*^24^,^28^,^29^. Genetic analyses of *Populus* hybrid zones in Europe have shown that, even in sympatric settings, parental species and their hybrids form three distinct ancestry groups^27^,^28^, with a strongly varied introgression from *P. tremula* into *P. alba* across marker loci^30^. Hybrids are genetically and phenotypically mainly intermediate between the parental species^25^,^27^. Few mature trees show backcross-like genotypes, and F1 hybrid genotypes that early studies suggested to be either very rare or absent^27^,^28^ actually prevail in *P.* × *canescens*^31^,^32^. Strong genomic discontinuities between hybrids and their parents, but weak reproductive isolation early in the life-cycle indicate that *Populus* hybrids act as strong genotypic filters^27^,^28^,^31^. A considerable amount of selection on some hybrid genotypes, such as backcrosses, might take place between the seedling stage and maturity^31^. The reduced fitness of hybrid seedlings, however, was often explained by the breakdown of co-adapted nuclear gene complexes but the plastid-nuclear interactions were overlooked. Especially, significant lineage disequilibria between the plastid variation and several nuclear loci have been detected in European hybrid zones^33^, suggesting non-random combination between plastid and nuclear genome in *Populus*. Thus, an alternative possibility is that incompatibility between the maternally-inherited plastid genome^34^ from one species and the biparentally-inherited nuclear genome from the other in hybrids have resulted in the “breakdown” of fitness, and finally exhibited significant linkage between the plastid genome and certain nuclear loci in the adult after selection against “incompatible” combinations. When the F1 hybrids in *P.* × *canescens* are as fit or even fitter than their parental species due to epistatic interactions within genomes^28^, declines in fitness can occur in the second (or later) hybrid generations due to the greater severity of incompatibilities between the plastid and homozygous nuclear genomes from different parental species^12^.

A natural hybrid zone between *P. alba* and *P. tremula* also occurs along the Irtysh River system in the Altai region of Northern Xinjiang, China^35^. Within this zone, the *P. alba* population is distributed in the lowland flood-plains of the Irtysh River, *P. tremula* populations are found in nearby mountainous regions, and their hybrids, *P.* × *canescens*, are prevalent along the tributaries of the Irtysh River, such as the Haba, Buerjin, and Buliezi Rivers. In this study, we described the genetic structure of individuals in the *P. alba* and *P. tremula* hybrid zone along the Irtysh River system, using both nuclear microsatellite and plastid DNA (i.e. chloroplast DNA, cpDNA) *trn*L-*trn*F sequences to test whether plastid-nuclear incompatibility contributes to maintaining the integrity of *Populus* species in the face of gene flow. More specifically, we aimed to answer the following questions, 1) Do *P. alba* and *P. tremula* maintain high nuclear and/or plastid divergence in the Irtysh River hybrid zone? 2) What is the genomic composition of hybrids in this zone? 3) Does significant linkage disequilibrium (LD) occur between plastid *trn*L-*trn*F variation and the nuclear microsatellite genotype? 4) What evolutionary processes may lead to the CND?

## Results

### Polymorphism at microsatellite loci

In each taxon, the number of alleles (*A*), variance in allele size (*Var*), expected heterozygosity (*H*_E_), and observed heterozygosity *(H*_O_) varied among 11 nuclear microsatellite loci (see Supplementary Table S1). Across all of these loci, both *H*_E_ and *H*_O_ for *P.* × *canescens* (0.678 and 0.649, respectively) were higher than those for *P. tremula* (0.586 and 0.524) and *P. alba* (0.360 and 0.430). Estimates of diversity [allelic richness (*A*_r_), *H*_E_, and *H*_O_] did not vary greatly across populations within each species, but *P. tremula* populations generally had higher diversity than *P. alba* populations (Table 1). In one *P. alba* population, *F*_IS_ was significantly greater than zero, and in two *P.* × *canescens* populations *F*_IS_ was significant lower than zero, displaying significant deviations from Hardy–Weinberg equilibrium (HWE) (Table 1). After sequential Bonferroni corrections were applied, significant LD was detected at 28 of 55 pairwise microsatellite locus comparisons in *P. tremula*, compared with only one pairwise locus comparison in *P. alba*. Significant LD in *P. tremula* mainly occurred in one population (Pt3). After excluding this population, significant LD was detected at only one pairwise microsatellite locus comparison in *P. tremula*. Significant LD was detected at 52 of 55 pairwise microsatellite locus comparisons in *P.* × *canescens*.

### Differentiation and structure

The differentiation between the two parental species was high across all microsatellite loci, as estimated by both *F*_ST_ (0.418, *p* < 0.001) and *R*_ST_ (0.564). Many loci exhibited very high differentiation, with seven of the 11 loci displaying both *F*_ST_ and *R*_ST_ values >0.3, only one having an *F*_ST_ < 0.1, and two having an *R*_ST_ < 0.1 (Table 2). The genetic differentiation between hybrid and parental species was lower than that between the parental species, but still moderately high and significant for most loci. However, the hybrid species showed a slightly higher genetic differentiation from *P. tremula (F*_ST_ = 0.182, *p* < 0.001; *R*_ST_ = 0.369) than from *P. alba (F*_ST_ = 0.120, *p* < 0.001; *R*_ST_ = 0.119), consistent with a stronger affinity of hybrids for *P. alba* than for *P. tremula* in the neighbor-joining tree based on the population allelic frequencies of the 11 microsatellite loci (see Supplementary Fig. S1). Within both parental species, nuclear differentiation among populations was low, but relatively higher values were found in *P. tremula (F*_ST_ = 0.105 and *R*_ST_ = 0.151) than that in *P. alba (F*_ST_ = 0.049 and *R*_ST_ = 0.089).

Analysis of molecular variance (AMOVA) showed that 42.3% of the nuclear microsatellite variation occurred between *P. alba* and *P. tremula*, resulting in a high statistic for interspecific genetic divergence (*F*_CT_ = 0.423). The proportion of variation among populations within each species was small (4.81%, *F*_SC_ = 0.081), while the proportion of variation within populations followed a complementary pattern (52.86%, *F*_ST_ = 0.571; Table 3).

**Figure 1:**
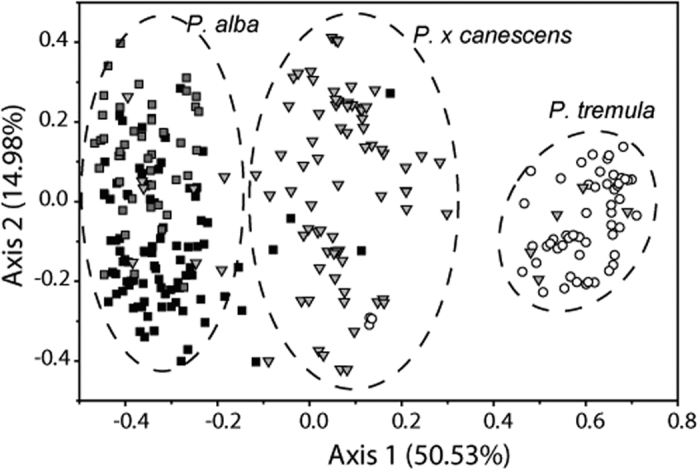
Principal coordinate plots of pairwise differentiation based on 11 nuclear microsatellites for 340 individuals of *P. alba, P. tremula*, and *P.* × *canescens*. Percentages of total variance explained by each axis are noted in brackets; black squares, *P. alba* from pure sites; grey squares, *P. alba* samples that coexisted with *P.* × *canescens*; triangles, *P.* × *canescens*; circles, *P. tremula*.

Principal coordinate analysis (PCoA) of the pairwise individual genetic distances is presented in Fig. 1. The first two PCo-axes of the plot accounted for >65% of the variation. The plot indicated that *P. alba, P. tremula*, and *P.* × *canescens* were mostly grouped separately in line with morphological classification in the field, except for several mismatches between hybrids and each parental species; the hybrid species were located between the two parental species, but had a stronger affinity to *P. alba* than to *P. tremula*. From the plot, compared with the two parental species, *P.* × *canescens* showed the largest genotypic variation among individuals, while variation among *P. alba* individuals was greater than that among *P. tremula* individuals, mostly coming from *P. alba* individuals in mixed locations.

### Nuclear admixture analysis

**Figure 2:**
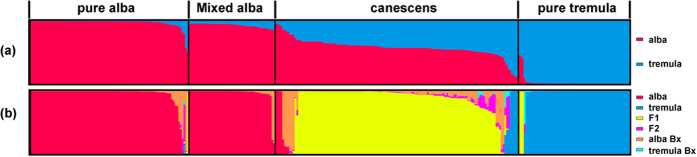
Bayesian clustering results of all sampled *Populus* individuals based on 11 nuclear microsatellite loci. (**a**) Individual assignment results using STRUCTURE software at *K* = 2. (**b**) Individual assignment results using NewHybrids software. In all diagrams, each column represents a single individual. The length of each colored segment is proportional to the posterior probability of assignment to *P. alba* or *P. tremula* in (**a**); or is proportional to the posterior probability of assignment to the corresponding genotypic class in (**b**).

STRUCTURE analysis classified all individuals from the Irtysh River hybrid zones into two clusters (*K* = 2, Supplementary Fig. S2). The majority of *P. alba* and *P. tremula* individuals from pure populations were classified into their respective clusters with high admixture coefficients (*Q* > 0.95), and only six *P. alba* and four *P. tremula* were classified as hybrids based on their *Q* values (Fig. 2a). The admixture proportions of *P. alba* individuals from *P.* × *canescens* mixed populations ranged from 0.849 to 0.957, with 23 of 49 individuals (46.9%) giving values >0.95. In contrast, a wide range of admixture proportions was found for *P.* × *canescens* hybrid morphotypes (*Q* ranged from 0.107 to 0.055, with a 95% confidence interval of *Q* = 0.348–0.825), indicating the presence of a wide range of hybrid generations and back-crossing to both parental species.

NewHybrids analysis also assigned most *P. alba* and *P. tremula* individuals to their respective clusters with high probabilities (>0.75; Fig. 2b). In addition, four *P.* × *canescens* individuals were identified as *P. alba* and five as *P. tremula*, with high probability (>0.98). Whereas individuals identified as hybrids in STRUCTURE analysis, including several *P. alba* and *P. tremula* and the majority of *P.* × *canescens*, were mostly assigned to hybrids. Using 0.75 as a threshold, 107 of the 136 *P.* × *canescens* individuals and one individual from a *P. tremula* population were identified as F1, eleven *P.* × *canescens* individuals and two *P. alba* individuals were identified as back-crossing to *P. alba*, and none were identified as F2 and back-crossing to *P. tremula*. Only two individuals were assigned to F2 and one was assigned to back-crossing to *P. tremula* with probabilities ~0.5.

### Plastid variation and cytonuclear association

**Figure 3:**
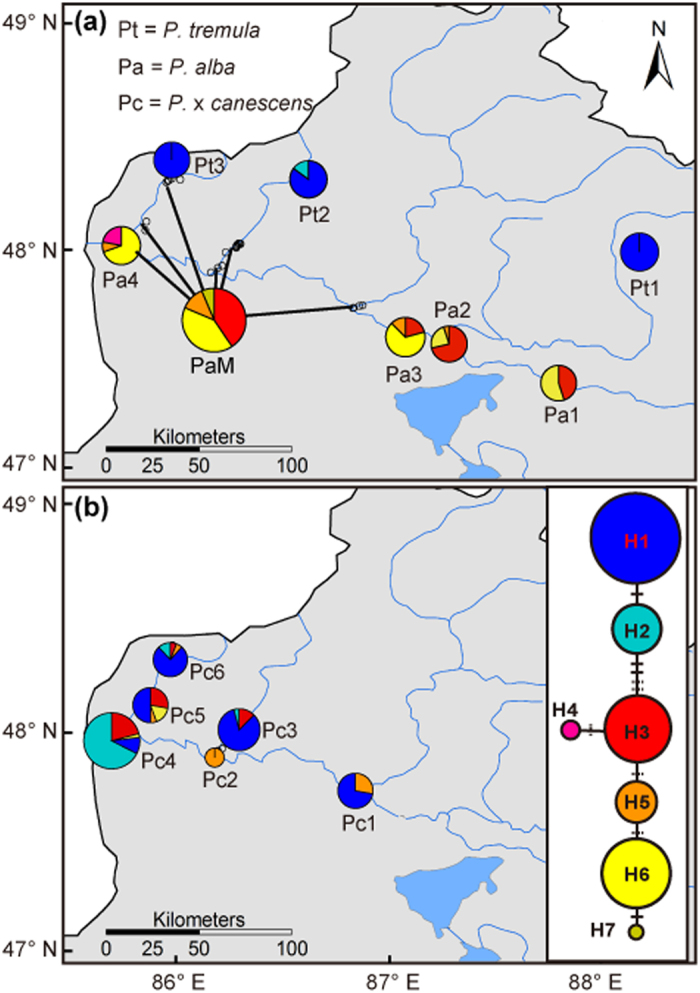
The *trn*L-*trn*F haplotype distribution for each *Populus* population in the Irtysh River hybrid zone. Distributions are shown separately in pure parental species (**a**) and in hybrid species (**b**). Circle sizes are proportional to the sample sizes of the populations. Right panel in (**b**) shows the network of haplotypes; perpendicular tick marks on the lines joining haplotypes represent the number of mutations: dotted ticks, indels; solid ticks, substitutions. The map was created using the ArcMap package in ArcGIS ver. 9.2 (http://www.esri.com/software/arcgis).

Single A/T indel variations in three poly-A/T regions (443, 790, and 930 bp) of *trn*L-*trn*F were excluded from analysis because of a potentially high rate of reverse mutation. A matrix of 1004-bp sequences from 325 individuals yielded four nucleotide substitutions and four indel variations, combining into seven haplotypes (H1–H7). Haplotype descriptions are listed in Supplementary Table S2. The sequences of these haplotypes have been deposited in GenBank (accession numbers KT851748- KT851754). Two of the haplotypes (H1 and H2) were detected in *P. tremula*, and the other five (H3–H7) in *P. alba* (Fig. 3a). Five of the haplotypes (two *P. tremula*, H1–H2, and three *P. alba*, H3, H5–H6) were detected in hybrid *P.* × *canescens* (Fig. 3b). Median-joining analysis resulted in a haplotype network with two major clades connected by three variations, one of which contained H1 and H2, while the other consisted of H3–H7 (Fig. 3b).

The gene diversity based on *trn*L-*trn*F for each *Populus* population varied from 0 (Pt1, Pt3, and Pc1) to 0.680 (Pc 4; Table 1). The total diversity in *P. alba (h*_T_ = 0.642) was much higher than that in *P. tremula (h*_T_ = 0.094), but slightly lower than in the hybrids (*h*_T_ = 0.692). AMOVA analysis showed that more than half of the *trn*L-*trn*F sequence variations (55.75%) occurred between *P. alba* and *P. tremula*, resulting in an *F*_CT_ reaching 0.558; only 8.50% of the variation occurred among populations within species (*F*_CS_ = 0.192), and 35.5% of the variation occurred within populations (*F*_ST_ = 0.643; Table 3).

The calculation of CND for the whole data set revealed clear deviation from a random distribution between plastid haplotypes and nuclear alleles (D, Table 4): *i.e.* pure nuclear *P. tremula* genotypes (TT) were significantly associated with the haplotypes of *P. tremula* (Ht), showing significant disequilibria involving homozygous genotypes (D1 and D3); the heterozygous genotypes were also positively associated with the haplotypes of *P. tremula* (D2) at seven of the eight examined nuclear loci. In the group of hybrids, significant positive CND was also found between plastid haplotype and nuclear alleles in three microsatellite loci (D), resulting in significant negative values of D3; a positive value of D1 was found to be significant at one locus and the values were very high at the other four loci (D1 = 1, Table 4), although they were not significant due to limited combination counts for statistics.

**Figure 4:**
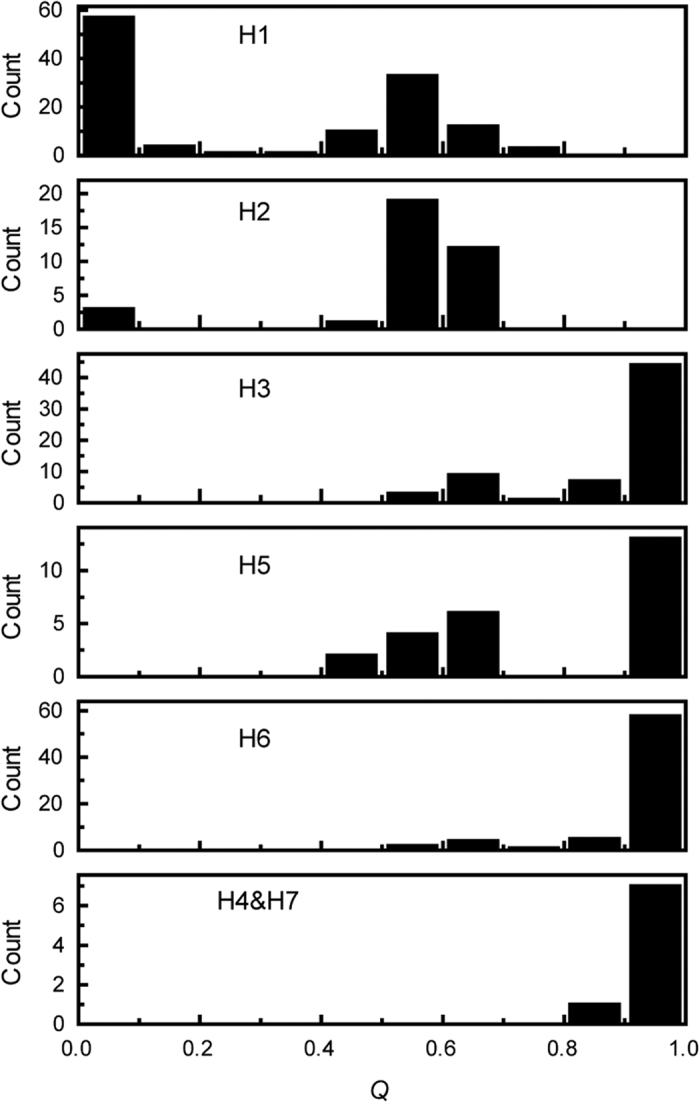
Distribution of estimates of admixture proportion in each *trn*L-*trn*F haplotype. The admixture proportion is based on the posterior probability of belonging to *P. alba (Q*) in STRUCTURE analysis (for *K* = 2) using the 11 nuclear microsatellites.

A simple test for correlation between *trn*L-*trn*F haplotypes and nuclear genomic composition (*Q* estimated by STRUCTURE analysis) yielded significant results both for all individuals (Spearman’s r = 0.794, P < 0.001) and after excluding *P. alba* and *P. tremula* populations (Spearman’s r = 0.675, P < 0.001). The distribution of admixture proportion (*Q*) in each haplotype showed that the range of *Q* varied greatly among haplotypes (Fig. 4). Haplotype H1 mainly occurred in pure *P. tremula* and hybrids, with a wide range of *Q* from 0 to 0.8; H2 mainly occurred in hybrids with *Q* ranging between 0.4 and 0.7, and was also found in several *P. tremula* individuals; H3 and H6 mainly occurred in pure *P. alba* and were found in a few hybrids with *Q* > 0.5. H5 occurred in pure *P. alba* and hybrids with a *Q* value between 0.4 and 0.7, while H4 and H7 only occurred in individuals showing *P. alba* ancestry.

## Discussion

*P. alba and P. tremula* are model species in which to study the later stages of tree speciation, and the maintenance of species identity with gene flow^36^. Our current analysis of the *P. alba* and *P. tremula* hybrid zone along the Irtysh River further supports the concept that high hybridization rates and appreciable hybrid fitness do not necessarily conflict with the maintenance of species integrity^31^. First of all, the results from both PCoA and STRUCTURE analysis showed that, in the Irtysh River region, *P. alba* and *P. tremula* both maintained their own species identity, although the hybrid *P.* × *canescens* was prevalent. The parental species and their hybrids were separated into three distinguishable groups in the PCoA plot, consistent with previous analysis of these three species in the European hybrid zones^27^,^28^. Meanwhile, consistent with the high interspecific *F*_ST_ values found in the European contact zone using similar markers (*F*_ST_ = 0.37)^37^, the mean genetic differentiation across the11 microsatellites was high between the two parental species in the Irtysh River hybrid zone (*F*_ST_ = 0.418 and *R*_ST_ = 0.564), reflecting their long divergence time and limited interspecific gene flow^28^. Furthermore, most of these microsatellite loci showed high interspecific differentiation, and the few loci that showed a low genetic differentiation may be a result of shared ancestry polymorphism or gene-flow between species^37^. Notably, STRUCTURE analysis that is based on the hypothesis of HWE found only two genetic clusters rather than three (Supplementary Fig. S2), suggesting that the hybrid *P.* × *canescens* has not yet formed a completely distinct gene pool. Notably, we found that population differentiation within parental species was relatively higher in *P. tremula (F*_ST_ = 0.105 and *R*_ST_ = 0.151) than that in *P. alba (F*_ST_ = 0.049 and *R*_ST_ = 0.089), which is contrary to the results from the European populations of these species, where much lower differentiation was always found among populations of *P. tremula*^28^. The higher differentiation of *P. tremula* in our current analysis was mainly caused by population Pt3, which was located at a lower altitude than other two *P. tremula* populations (see Supplementary Table S3). In this population, significant LD was detected at 20 of 55 pairwise microsatellite locus comparisons, indicating assortative mating or selection on certain genotype combinations. Further analysis of this population is needed to understand the adaptive evolution of *P. tremula*.

Consistent with the results of a recent study from the European hybrid zones^32^, our current analysis also found that F1 hybrids prevailed in the Irtysh River hybrid zone; however, due to differences in analytical methods, the hybrid *P.* × *canescens* had previously been considered to be highly recombinant^25^,^27^,^28^. Their prevalence in both of these geographically distant hybrid zones are unlikely to be strongly affected by stochastic or locality-specific events, but rather reflect the high fitness of such genomic patterns of hybrids due to heterosis^28^,^31^,^32^. Although the wide range of *Q* values in STRUCTURE analysis based on nuclear microsatellites indicated that hybrids could back-cross towards both parental species in the Irtysh River hybrid zone (Fig. 2a), NewHybrids analysis found almost no F2 hybrids and back-cross hybrids towards *P. tremula* (Fig. 2b). It is possible that the NewHybrids analysis could not discriminate between pure *P. tremula* and back-crosses due to limited microsatellite loci^38^, so some hybrids with an ancestry close to *P. tremula* were assigned to the pure species (Fig. 2b). Meanwhile, a low frequency of back-cross hybrids was evident from the results of both STRUCTURE and NewHybrids analysis (Fig. 2). We suggest that strong viability selection from seedling to adult may be responsible for the sparseness of later-generated hybrids and this may contribute to the maintenance of high species differentiation with hybridization in *Populus*^31^,^32^. Especially, weak reproductive isolation between the two species and their hybrids was suggested by the presence of the full range of hybrids in seedlings in the European hybrid zones^31^, and have been found in our hand-pollinated hybridization as well (unpublished data). However, in contrast to those hybrids back-crossing toward *P. tremula*, more back-cross hybrids toward *P. alba* were detected in NewHybrids analysis. This is reasonable, as *P.* × *canescens* coexisted with *P. alba*, and so had a higher possibility of back-crossing with it. The preponderance of F1 hybrids was also supported by the PCoA plot (Fig. 1), in which most hybrids were intermediate between the two parental species. While estimation of genetic differentiation, the PCo plot, and the population neighbor-joining tree all suggested that hybrids had a slightly stronger affinity to *P. alba* than to *P. tremula*, reflecting preferential introgression back-crosses towards *P. alba*^24^,^27^,^28^. It is also possible that our inferred F1 hybrids carried older traces of recombination not visible with limited genetic markers in current study; whereas older admixture pulses were detected in European hybrid zone using high-density single-nucleotide polymorphisms (SNPs)^32^.

A previous study on hybrid zones of *P. tremula* and *P. alba* in the Danube valley found that the hybrid *P.* × *canescens* preferentially carried the plastid DNA of *P. alba*, indicating that hybridization occurred preferentially *via P. tremula* pollen and *P. alba* seed parents^24^. Lexer *et al*.^24^ suggested that this unidirectional pattern is facilitated by high levels of pollen *versus* seed dispersal in *P. tremula* and by great ecological opportunity in the lowland flood-plain forest near *P. alba* seed parents. However, our current analysis showed that *P.* × *canescens* carried the plastid DNA of both *P. tremula* and *P. alba*, and even more hybrids carried the plastid DNA of the former, reflecting positive associations between the heterozygous genotypes and haplotypes of *P. tremula* (D2, Table 4). This means that, in the Irtysh River valley, hybridization could occur *via P. alba* pollen and *P. tremula* seed parents, as well as *via P. tremula* pollen and *P. alba* seed parents. During the sampling, we found that in this zone, hybrids mostly occurred in the flood-plains of tributaries or the flood-plains where tributaries join the main river; only a few occurred in the flood-plain of the main river, where *P. alba* was prevalent. Thus, it is possible that the hybrid seeds from *P. tremula* that grow in the upland are dispersed to the lowland flood-plain by wind or by the flow of melt-water along the tributaries of the Irtysh River every spring^35^.

Unlike potential nuclear introgression, the plastid *trn*L-*trn*F sequence was completely distinct between the two parental species in the Irtysh River hybrid zone (Fig. 3a). Significant CNDs across all loci were found in the whole dataset, demonstrating a non-random association of the nuclear genome with the plastid genomes in this *Populus* hybrid zone (Table 4). Departures from random associations between cytoplasmic and nuclear genetic markers in hybrids can reflect the migration of parental genotypes into the hybrid populations, assortative mating among similar genotypes, selection due to cytonuclear interactions, or cytoplasmic male sterility (see references in Cruzan & Arnold^23^). Factors like assortative mating and migration can generate a CND *de novo*, and they are likely to affect all chromosomes^39^. Strong CND observed consistently across loci for the whole data set might be driven by migration and assortative mating of parental genotypes in the large hybrid zone. Especially, *P. alba* and *P. tremula* are distributed separately in river basins and in mountainous regions, respectively, thus mating tends to occur within each species.

However, within the hybrid *P.* × *canescens* group, the plastid of *P. tremula* was also significant associated with nuclear alleles of *P. tremula* in several but not all microsatellite loci (Table 4). Significant CND between the plastid and a few nuclear loci in *P.* × *canescens* has also recently been found in the European hybrid zone^33^. The contribution of parental conspecifics to CND by migration could be neglected, as only hybrids were included in the analysis; assortative mating, however, is not possible for wind-pollinated *Populus* when the flowering times of the two parental species largely overlap^33^ and hybrids with different haplotypes always coexisted in the same forest (Fig. 3b). It is possible that such a CND pattern in hybrids is caused by the action of selection on interspecific combinations of cytoplasmic and nuclear genotypes due to incompatibility. Unlike CND caused by assortative mating and migration that affect all chromosomes, that caused by selection is more locus-specific^22^; only the microsatellite markers linked to certain genes involved in cytonuclear processes might cause the observed disequilibria, especially in early-generation hybrids carrying relatively large chromosome blocks inherited from each parental species^33^.

Selection against certain hybrid classes has been identified in the European *Populus* hybrid zone^31^,^32^, and was also supported by the low frequency of post-F1 hybrids in the current zone. More important, the significant association between plastid haplotypes and the proportion of nuclear admixture in hybrids (Spearman’s r = 0.675, P < 0.001) suggested that the haplotype distribution in different hybrid classes was also non-random: the *P. tremula* haplotypes tended to occur in hybrids with a reduced genomic composition of *P. alba (Q *< 0.8); *P. alba* haplotypes only occurred in hybrids with a reduced genomic composition of *P. tremula (Q *> 0.4), demonstrating a pattern of interspecific plastid-nuclear incompatibility (Fig. 4). Therefore, we infer that the “breakdown” of fitness by incompatibility might have resulted in selection against later-generated hybrids carrying interspecific combinations of cytoplasmic and nuclear genotypes and finally exhibited significant linkage between the plastid genome and certain nuclear loci.

What to mention, a recent study from European hybrid zones inferred that cytonuclear interactions were less likely to contribute to genomic isolation of these poplar in three studied localities, where cytoplasms of each species appeared to combine freely with different nuclear genomes^32^. Consistent with results of their earlier study^24^, Christe *et al*.^32^ found that hybrids in these localities tended to carry the plastid genome of their spatially closer parent. It is reasonable, as the plastid genome of poplar is seed-mediated and seeds are less mobile than their pollen. However, the spatial distribution of hybrids could not fully explain the observed plastid-nuclear pattern in the Irtysh River hybrid zone, where *P.* × *canescens* individuals (Pc) coexisted with *P. alba* (PaM in Fig. 3a) and carried haplotypes of either parents, no matter where they located (Fig. 3b). Of cause, to confirm the role of cytonuclear interactions on the observed plastid-nuclear pattern of hybrids, more hybrid samples are needed to exclude the influence of a spatial distribution, especially those locate close to *P. tremula,* and more markers are necessary to scale the nuclear composition of hybrids more accurately.

In all, our results showed that plastid-nuclear incompatibility might have established hybridization barriers between parental species and hybrids in the Irtysh River hybrid zone and finally influenced the extent and pattern of introgression between *P. tremula* and *P. alba*. However, to further assess the role of plastid-nuclear incompatibility in the speciation of *Populus*, more genetic information on the plastid genomes of *P. tremula* and *P. alba* are needed to allow comparison of the adaptive divergence of related proteins between species. Also, hand-pollination studies are needed to compare the hybrid variegation among different plastid and nuclear genome combinations. In the current analysis, we only chose markers within the plastid genome, and thus only considered the association between plastid and nuclear genomes. Meanwhile, the influence of the mitochondrial genome cannot be excluded, as it is also maternally inherited in *Populus* and thus linked to the plastid genome^34^,^40^.

## Materials and Methods

### Study sites and sampling

The two parental species, *P. alba* (white poplar) and *P. tremula* (European aspen), and the hybrid *P.* × *canescens* (gray poplar) were sampled from the Irtysh River system from 85°E to 88°E, including the main Irtysh River and three tributaries: the Haba, Buerjin, and Buliezi Rivers. In this region, pure *P. alba* populations were mainly found along the main Irtysh River; pure *P. tremula* populations were located in mountainous regions near tributaries; and *P.* × *canescens* prevailed along the tributaries with a few coexisting *P. alba*.

Individuals were classified into different taxa in the field based on leaf morphology, such as shape and abaxially white tomentose, following the Flora of China^41^. In total, 63 *P. tremula* individuals were sampled from three pure populations (Pt1–3) and 90 *P. alba* individuals were sampled from four pure populations (Pa1–4). In addition, 136 *P.* × *canescens* individuals and 49 *P. alba* individuals (PaM) that coexisted along the three river tributaries were sampled from 28 sites (see Supplementary Table S3). For analysis, hybrid individuals from these sites were clustered into six populations by combining nearby sites (Pc1–6, see Supplementary Fig. S3). The sampled trees were at least 50 m distant from each other. Leaves were dried using silica gel and then taken back to the laboratory for DNA extraction.

### Microsatellite marker procedure

The samples were screened for variation at 11 nuclear microsatellite loci supplied by the International Populus Genome Consortium (http://www.ornl.gov/sci/ipgc/ssr_resource.htm), see Supplementary Table S4 for primer details. The 11 microsatellites were amplified by the polymerase chain reaction (PCR) following the methods described by He *et al*.^42^, with each forward primer labeled with one of the fluorescent dyes 6-FAM, HEX, or TAMRA (Sangon Biotech, Shanghai, China).

The microsatellite genotypes of all loci were resolved on an ABI 3130xl automated sequencer (Life Technologies, Foster City, CA), making use of the different fluorescent dyes and size differences between loci for multiplexing. Molecular sizes in base pairs were determined using the GENESCAN-500 ROX size standard. The microsatellite genotyping was subsequently analyzed using GENEMAPPER software version 3.7 (Life Technologies) and visually checked twice. Each set of 48 reactions included a positive (known genotype) and a negative (water) control carried from PCR through to the final automated sequencer analysis of microsatellites.

### Plastid sequence procedure

The plastid intergenic spacer *trn*L-*trn*F region was amplified using the primers *trn*LF-f (5′-ATTTGAACTGGTGACACGAG-3′) and *trn*LF-r (5′-CGAAATCGGTAGACGCTACG-3′), which were improved from the original of Taberlet *et al*.^43^, based on the complete genome of the *P. trichocarpa* chloroplast (GenBank accession number NC_009143^44^). PCR was performed in a 25-μL volume, containing 10 mM Tris-HCl (pH 8.0), 0.2 μM of each dNTP, 1.5 mM MgCl_2_, 0.16 μM of each forward and reverse primer, 1 U *Taq* DNA polymerase (TaKaRa, Tokyo, Japan), and 1 μl (10–50 ng) genomic DNA. PCRs were performed in an Eppendorf Mastercycler, programmed for initial denaturing at 94 °C for 4 min, followed by 30 cycles of denaturing for 45 s at 94 °C, annealing at 60 °C for 45 s, and extension for 1 min and 20 s at 72 °C. A final extension followed at 72 °C for 8 min. PCR products were purified with a Quick PCR Purification Kit (Tiangen, Beijing, China), and then all DNA sequencing was performed using an ABI Prism Bigdye^TM^ terminator cycle sequencing ready reaction kit (Life Technologies, Foster City, CA). The reaction mixtures were analyzed on an ABI 3130xl automated sequencer (Life Technologies).

### Nuclear data analysis

#### Nuclear genetic diversity and differentiation

In order to characterize the microsatellite loci in the two study species and their hybrids, the number of alleles (*A*), variance in allele size (*Var*), expected heterozygosity (*H*_E_), and observed heterozygosity (*H*_O_) were calculated for each locus. In each population, *Var*, allelic richness (*A*_r_), *H*_E_, and *H*_O_ were calculated across all microsatellite loci. All analyses were performed using the MSA program^45^. The inbreeding coefficient *F*_IS_ was calculated using FSTAT^46^ and the significance of *F*_IS_ was tested for departures from HWE for each population after Bonferroni correction. LD among microsatellite loci was analyzed for each species using the log-likelihood ratio statistic (G-test) implemented in Genepop 4.3^47^ with the default set of Markov chain parameters and the nominal level of statistical significance set at 0.01.

The genetic differentiation between species or among populations within species was estimated by *F*_ST_^48^ and *R*_ST_^49^ for the 11 microsatellite loci using the program FSTAT 2.9.3^46^. The significance of *F*_ST_ was tested by comparison of the observed *F*_ST_ with a distribution of *F*_ST_ under the hypothesis of no genetic structure, obtained by means of 5,000 random permutations of individuals between species or among populations.

AMOVA in Arlequin 3.11^50^ was used to obtain F-statistics for nuclear microsatellite markers between the two parental species. We tested the hierarchies “among species”, “among populations within species”, and “within populations” using only pure populations of *P. alba* and *P. tremula*.

PCoA analysis was performed for microsatellite data sets to calculate principal co-ordinates from pairwise Euclidian distance estimates between individual genotypes. Analyses were executed in GenAlEx6^51^. The first two axes were plotted graphically with Origin 7.5 (OriginLab, Northampton, MA).

#### Nuclear admixture analysis

To identify the possible genetic structure and admixture of individuals in the hybrid zone, we used a model-based Bayesian approach implemented in STRUCTURE ver. 2.3.3^52^,^53^. The program was run, without prior population information, under the admixture model allowing for correlated allele frequencies. Ten independent replicates for each *K* were analyzed for *K* = 1–7, setting burn-in and run lengths of 500 000 and 2 000 000 iterations, respectively. The final posterior probability of *K, Pr(X|K*), and delta*K* (Δ*K*), where the modal value of the distribution is located at the real *K*^54^, were used to determine the most likely number of clusters.

The method developed by Anderson and Thompson^55^ and performed in NewHybrids was used to achieve a more detailed analysis of admixture proportions and hybrid ancestry by inferring the posterior probability assignment (*Q*) of each sampled individual to one of six genotype frequency classes: *P. tremula, P. alba*, F1 (first generation hybrids), F2 (second generation hybrids, crossing of F1 × F1), B × *tremula* (back-cross of F1 hybrids towards pure *P. tremula*) and B × *alba* (back-cross of F1 hybrids towards pure *P. alba*). The program was run with the nuclear microsatellite data using 50 000 burn-in iterations followed by 500 000 Markov chain Monte Carlo iterations using default priors for allele frequencies and mixing proportions.

### Plastid DNA analysis

The sequences of *trn*L-*trn*F were first aligned with Clustal X^56^ and then manually adjusted. A matrix of combined sequences was constructed for the 325 individuals that we examined and different plastid sequences were identified as haplotypes. A median-joining network^57^ was constructed between haplotypes using the program Network ver. 4.6.1.3. (www.fluxus-engineering.com).

Gene diversity, the probability that two randomly-chosen homologous sites are different, was analyzed for both populations and species using ARLEQUIN ver. 3.1^58^. With the same program, AMOVA was used to obtain F-statistics for among species, among populations within species, and within populations, using plastid sequences of only pure *P. alba* and *P. tremula* populations.

Plastid-nuclear LD was estimated between each nuclear microsatellite locus and the plastid *trn*L-*trn*F locus by testing departures from random cytonuclear associations^22^ using the CNDm program^59^. The analyses were carried out by encoding nuclear markers in the form of synthetic alleles (T, alleles typical of *P. tremula*; A, alleles typical of *P. alba*) and by encoding plastid DNA alleles as synthetic haplotypes (Ht, haplotypes typical of *P. tremula*; Ha, haplotypes typical of *P. alba*). Species-specific nuclear alleles were defined based on both clear frequency differences and diagnostic alleles. The loci GCPM1063, GCPM1065, and GCPM1353, whose common alleles did not show clear frequency differences between the two species, were excluded from analysis.

The normalized CND was calculated for the whole dataset and for the hybrid *P.* × *canescens* only, following Asmussen and Basten^60^ for allelic and genotypic associations, and significance levels were tested using Fisher’s exact test. Significantly positive and negative values of CND indicate positive and negative associations between nuclear and cytoplasmic genomes, respectively. Significant disequilibria involving heterozygous nuclear loci (TA) – represented by D2 (TA/Ht) – point to nonrandom mating in hybrid zones and unidirectional hybridization, whereas significant disequilibria involving homozygous genotypes D1 and D3 (involving nuclear genotypes TT and AA, respectively) point to barriers to introgression, effectively maintaining species integrity in the face of gene flow^39^.

To test for a possible role of plastid-nuclear incompatibilities in determining the patterns of introgression across the hybrid zone, the proportion of nuclear admixture (*Q*) estimated by the STRUCTURE program at *K* = 2 and plastid haplotype data were compared in a simple way: nonparametric Spearman rank correlations were used to test for a possible association between nuclear and plastid genomic composition using SPSS (SPSS Inc., Chicago, IL). The test was done both for all individuals and for the hybrid *P.* × *canescens* only. The distribution of nuclear admixture proportions was demonstrated for each *trn*L-*trn*F haplotype with Origin 7.5 (OriginLab, Northampton, MA).

## Additional Information

**How to cite this article**: Zeng, Y.-F. *et al*. Genetic structure of *Populus* hybrid zone along the Irtysh River provides insight into plastid-nuclear incompatibility. *Sci. Rep.* **6**, 28043; doi: 10.1038/srep28043 (2016).

## Supplementary Material

Supplementary Information

## Figures and Tables

**Table 1:** Genetic variability in each sampled *Populus* population.

	Microsatellite							*trn*L-*trn*F	
	*N*	*A*	*A*_R_	*Var*	*H*_O_	*H*_E_	*f*	*N*_H_ (sample size)	*Gd*
Pt1	23	5	4.62	22.4	0.479	0.540	0.115	1 (23)	0.000
Pt 2	19	4	4.28	22.4	0.525	0.560	0.065	2 (20)	0.268
Pt 3	21	4	4.23	48.1	0.573	0.531	−0.081	1 (21)	0.000
Pa1	20	4	3.45	33.5	0.310	0.396	0.223^*^	2 (20)	0.521
Pa2	20	3	3.08	32.7	0.373	0.402	0.076	3 (20)	0.416
Pa3	25	4	3.33	30.6	0.357	0.391	0.088	3 (24)	0.518
Pa4	25	5	3.92	38.9	0.396	0.458	0.139	3 (23)	0.482
PaM	49	3	2.63	27.0	0.369	0.366	−0.005	4 (49)	0.662
Pc1	6	2	NA	11.4	0.727	0.420	−0.868^*^	1 (6)	0.000
Pc2	25	5	4.46	54.4	0.668	0.636	−0.052	3 (24)	0.301
Pc 3	44	4	3.21	43.3	0.787	0.555	−0.427^*^	3 (43)	0.494
Pc 4	21	5	4.89	53.8	0.575	0.583	0.013	4 (18)	0.680
Pc 5	18	5	4.51	63.9	0.658	0.670	0.018	4 (17)	0.419
Pc 6	22	4	3.43	39.4	0.557	0.579	0.040	2 (19)	0.410

*A*, number of alleles; *A*_R_, allelic richness; *Var*, variance in allele size; *H*_O_, observed heterozygosity; *H*_E_, expected heterozygosity; *f*, inbreeding coefficient; *N*_H_, number of plastid DNA haplotypes found in each population; *Gd*, gene diversity analogous to *H*_E_; ^*^P < 0.05.

**Table 2:** Pairwise genetic differentiation between two parental species and their hybrids.

Locus	*trem vs alba*		*trem vs cans*		*alba vs cans*	
	*F*_*ST*_	*R*_*ST*_	*F*_*ST*_	*R*_*ST*_	*F*_*ST*_	*R*_*ST*_
GCPM1063	0.217^*^	0.489	0.073^*^	0.147	0.048^*^	0.191
GCPM1065	0.158^*^	0.199	0.083^*^	0.057	0.020^*^	0.047
GCPM114	0.601^*^	0.671	0.213^*^	0.206	0.190^*^	0.241
GCPM1158	0.720^*^	0.633	0.284^*^	0.276	0.241^*^	0.173
GCPM124	0.444^*^	0.593	0.183^*^	0.142	0.125^*^	0.199
GCPM1252	0.135^*^	0.081	0.051^*^	0.108	0.173^*^	−0.002
GCPM1255	0.857^*^	0.804	0.375^*^	0.330	0.324^*^	0.306
GCPM1260	0.317^*^	0.462	0.142^*^	0.227	0.069^*^	0.227
GCPM1274	0.368^*^	0.775	0.343^*^	0.741	0.000	−0.008
GCPM1353	0.095^*^	0.116	0.071^*^	0.028	0.004^*^	0.027
GCPM139	0.360^*^	0.009	0.177	0.143	0.129^*^	0.045
All	0.418^*^	0.564	0.182^*^	0.369	0.120^*^	0.119

*trem, Populus* *tremula*; *alba, P. alba; cans, P. × canescens*. ^*^P <0.05.

**Table 3:** Analysis of molecular variance (AMOVA) for nuclear microsatellite and plastid DNA data at three hierarchical levels.

	Microsatellites					Plastid DNA				
	d.f.	Sum of squares	Variation (%)	F-statistic	P	d.f.	Sum of squares	Variation (%)	F-statistic	P
Between species	1	242.17	42.34	*F*_*CT*_ = 0.423	<0.001	1	485.168	55.75	*F*_CT_ = 0.558	<0.001
Among populations within species	5	48.631	4.81	*F*_CS_ = 0.083	<0.001	5	123.549	8.5	*F*_CS_ = 0.192	<0.001
Within populations	299	585.385	52.86	*F*_ST_ = 0.471	0.025	142	579.122	35.75	*F*_ST_ = 0.643	0.026

Variance analysis only included the four pure *Populus alba* and three pure *P. tremula* populations.

**Table 4:** Cytonuclear disequilibria in Irtysh River hybrid zones of *Populus tremula* and *P. alba*.

Locus	Whole data set				*P.* × *canescens*			
	D1 (TT/Ht)	D2 (TA/Ht)	D3 (AA/Ht)	D (T/Ht)	D1 (TT/Ht)	D2 (TA/Ht)	D3 (AA/Ht)	D (T/Ht)
GCPM114	1.000^***^	0.471^***^	−0.844^***^	0.893^***^	1.000	0.331^**^	−0.517^***^	0.564^*^
GCPM1158	0.971^***^	0.393^***^	−0.837^***^	0.882^***^	1.000	−0.147	−0.074	0.206
GCPM124	0.968^***^	0.433^***^	−0.774^***^	0.833^***^	1.000	0.001	−0.122	0.217
GCPM1252	0.835^***^	0.636^***^	−0.745^***^	0.777^***^	0.480^**^	−0.342	−0.582^*^	0.516^***^
GCPM1255	0.970^***^	0.307^***^	−0.884^***^	0.914^***^	0.355	0.220	−0.457	0.434
GCPM1260	0.506^***^	0.250^***^	−0.818^***^	0.654^***^	0.052	0.040	−0.209	0.118
GCPM1274	0.839^***^	−0.043	−0.632^***^	0.721^***^	0.417	0.599	−0.494	0.466
GCPM139	1.000^***^	0.659^***^	−0.823^***^	0.874^***^	1.000	0.554^***^	−0.617^***^	0.635^***^

D1, D2, D3, genotypic disequilibria; D, allelic disequilibria; TT, nuclear genotypes typical of *P. tremula*; TA, nuclear genotypes heterozygous for T and A alleles; AA, nuclear genotypes typical of *P. alba*; Ht, plastid DNA haplotype typical of *P. tremula*; Fisher’s exact test: ^*^0.01 < P  < 0.05, ^**^0.001 < P < 0.01, ^***^P < 0.001.
