# Supplementary material for: Genetic structure of Populus hybrid zone along the Irtysh River provides insight into plastid-nuclear incompatibility
Source: Sci Rep. 2016 Jun 16;6:28043. doi: 10.1038/srep28043 (PMC4910079; doi:10.1038/srep28043)
Supplement: Supplementary Information [file srep28043-s1.pdf]

Genetic structure of *Populus* hybrid zone along the Irtysh River provides insight into plastid-nuclear incompatibility

Authors and Affiliations

Yan-Fei Zeng[1], Jian-Guo Zhang[1,2*], Ai-Guo Duan[1], Bawerjan Abuduhamiti[3]

[1]State Key Laboratory of Tree Genetics and Breeding, Key Laboratory of Tree Breeding and Cultivation, State Forestry Administration, Research Institute of Forestry, Chinese Academy of Forestry, Beijing, China

[2]Collaborative Innovation Center of Sustainable Forestry in Southern China, Nanjing Forestry University, Nanjing, China

[3]Forest Research Institute of the Altai Region, Xinjiang, China

[*]Corresponding author:

Jian-Guo Zhang: zhangjg@caf.ac.cn

Supplementary information

Supplementary Tables

Table S1. Genetic variability and population differentiation at 11 microsatellite loci in *P. alba*, *P. tremula*, and their hybrid (*P.* × *canescens*).

	P. alba						*P.* × *canescens*						*P. tremula*					
Locus name	A	*Var*	H_O	H_E	F_{ST}	R_{ST}	A	Var	H_O	H_E	θ	R_{ST}	A	*Var*	H_O	H_E	F_{ST}	R_{ST}
GCPM1063	5	4.5	0.435	0.543	-0.001	0.036	11	16.3	0.728	0.771	0.123*	0.192	11	24.3	0.937	0.836	0.025*	0.046
GCPM1065	5	1.3	0.108	0.219	0.011	-0.017	5	4.6	0.485	0.467	0.126*	0.068	6	7.3	0.619	0.713	0.054*	0.108
GCPM114	4	2.8	0.050	0.050	-0.003	0.004	6	120.0	0.809	0.622	0.093*	0.075	9	128.4	0.433	0.787	0.232*	0.237
GCPM1158	8	91.7	0.250	0.242	0.036*	0.064	9	119.6	0.919	0.624	0.073*	0.127	7	13.5	0.238	0.263	0.045*	0.077
GCPM124	7	9.1	0.415	0.449	0.123*	0.084	6	33.0	0.909	0.673	0.088*	0.036	6	21.0	0.667	0.605	0.018	0.062
GCPM1252	7	63.1	0.314	0.521	0.098*	0.107	10	38.8	0.209	0.774	0.239*	0.301	8	18.3	0.603	0.680	0.052*	0.153
GCPM1255	7	3.0	0.167	0.196	-0.023	0.007	7	6.4	0.904	0.523	0.008	0.032	5	1.0	0.095	0.123	-0.009	0.056

GCPM1260	11	19.8	0.524	0.796	0.054*	-0.012	9	10.3	0.735	0.684	0.078*	0.009	3	1.0	0.365	0.331	0.083*	-0.011
GCPM1274	6	57.2	0.553	0.582	0.008*	0.015	5	79.2	0.326	0.603	0.095*	0.077	7	56.8	0.510	0.591	0.065*	0.044
GCPM1353	9	4.6	0.691	0.575	-0.003	0.015	10	47.8	0.711	0.619	0.093*	0.037	9	99.6	0.803	0.812	0.135*	0.168
GCPM139	10	141.7	0.457	0.557	0.149*	0.167	8	106.4	0.709	0.719	0.147*	0.211	8	9.7	0.492	0.706	0.263*	0.009
Mean	7	36.3	0.360	0.430	0.049*	0.089	8	53.0	0.677	0.643	0.111*	0.123	7	34.6	0.524	0.586	0.105*	0.151

A, number of alleles; *Var*, variance in allele size; H_O, observed heterozygosity; H_E, expected heterozygosity; *P <0.01

Table S2. *Trn*L-*trn*F sequence variations of *Populus* in the Irtysh River hybrid zone identifying seven haplotypes

Haplotype	61	419-433	512	692-708	814-818	832	864	972
H1	A	#	A	-	-	C	T	C
H2	A	-	A	&	-	C	T	C
H3	A	#	A	&	-	C	T	C
H4	-	-	G	-	-	C	C	T
H5	-	-	A	-	-	C	C	T
H6	A	-	A	&	-	T	T	C
H7	A	#	A	-	@	C	T	C

#, TTCAAATAATAATAAA

&, TGTTTATCTTATTCTCT

@, ATAGA

Table S3. Sampling locations and classification.

Site	Population	Latitude (N)	Longitude (E)	Altitude (m)	*P. tremula*	*P. alba*	*P.× canescens*
1	Pt1	47°58′08″	88°11′18″	1305	23	0	0
2	Pt2	48°17′84″	86°36′27″	1154	19	0	0
3	Pt3	48°22′46″	85°56′35″	744	21	0	0
4	Pa1	47°21′50″	87°48′19	500	0	20	0
5	Pa2	47°33′26″	87°15′33″	475	0	20	0
6	Pa3	47°34′59″	87°03′26″	471	0	25	0
7	Pa4	48°00′03″	85°42′11″	424	0	25	0
8	Pc1	47°43′04″	86°49′25″	469	0	0	6
9	Pc1	47°43′00″	86°49′12″	481	0	0	11
10	Pc1	47°43′46″	86°52′04″	474	0	0	5
11	Pc1	47°43′33″	86°51′06″	480	0	4	0
12	Pc2	47°52′50″	86°07′53″	438	0	7	0
13	Pc2	47°54′03″	86°09′53″	447	0	2	0
14	Pc2	47°54′40″	86°11′09″	445	0	0	6
15	Pc3	47°58′23″	86°12′14″	454	0	0	5
16	Pc3	48°00′50″	86°16′36″	487	0	0	2
17	Pc3	47°59′46″	86°15′15″	472	0	0	2
18	Pc3	47°59′52″	86°15′18″	470	0	0	5
19	Pc3	48°00′04″	86°15′24″	472	0	0	2

20	Pc3	48°00′10″	86°15′16″	476	0	0	3
21	Pc3	48°00′34″	86°15′32″	482	0	0	2
22	Pc3	48°00′54″	86°15′58″	493	0	0	4
23	Pc3	48°00′43″	86°16′10″	481	0	2	0
24	Pc4	48°00′12″	85°42′09″	417	0	1	4
25	Pc4	48°00′44″	85°42′31″	420	0	3	20
26	Pc4	48°01′03″	85°42′43″	421	0	2	14
27	Pc4	48°01′20″	85°43′08″	421	0	5	6
28	Pc5	48°06′56″	85°49′10″	469	0	6	10
29	Pc5	48°04′26″	85°48′53″	457	0	1	11
30	Pc6	48°18′52″	85°56′29″	528	0	8	0
31	Pc6	48°18′52″	85°56′29″	526	0	4	2
32	Pc6	48°18′34″	85°58′58″	520	0	0	8
33	Pc6	48°18′22″	85°55′39″	506	0	0	3
34	Pc6	48°17′51″	85°54′56″	510	0	4	2
35	Pc6	48°18′11″	85°55′23″	518	0	0	3
	All				63	139	136

Table S4. Description of the 11 microsatellite loci analyzed in the current study

Name	Left Primer	Right Primer	Allele range (bp)	Motif	Chr.[#]	Labels[&]
GCPM1063	AGTTAATTGCGCATGTTCTT	AAACAAACTCCAGCAAACAT	136-178	ca	5	FAM
GCPM1065	TGCAATCATATATTCCTCCC	ATAAAATTACTGCGTGCCAT	146-164	ac	6	HEX
GCPM114	TTAGCCATTGGATTTCATTT	CATTGCACTCTCACACATTC	104-143	ttc	10	TAMRA
GCPM1158	ATGCACTTCCTTCCAAATTA	ATCAGTTCCTTCAGCTTCAa	202-262	ctg	2	HEX
GCPM124	TTTGAGCACTTCAACTACCA	TGTCTTCCCTTAGTCACCAC	175-202	cac	1	HEX
GCPM1252	AGCGTCTCAATGTTTTGTTT	TTTGCTTCAGGTTTATTTCC	114-138	aata	8	TAMRA
GCPM1255	GAACCTTAAAACCAGaaCCC	gagccacagaAatActgctc	163-191	ag	5	FAM
GCPM1260	CACAGGAACCTGGTTATCAT	CTGGCATTCCTTCTAAGCTA	131-163	tg	7	TAMRA
GCPM1274	GCCTGATACTTGTGGACCTA	cccGTATaaTATGatgatcca	162-200	ttat	1	HEX
GCPM1353	GAAAACTGATTCCTGATTCG	CAAGAATCAATGCATGTCTG	134-178	at	13	FAM
GCPM139	ATGACATGACATGATTGGAA	CTTCTGCTGGAAGAAGAAAA	189-239	gt	6	FAM

All markers are from http://www.ornl.gov/sci/ipgc/ssr_resource.htm; [#]Chr., chromosome location for each microsatellite locus; [&]forward primers were modified at the 5’ end with a fluorescent label: HEX (green), 6-FAM (blue), or TAMRA (yellow) (see Materials and Methods, PCR amplification).

Supplementary Figures

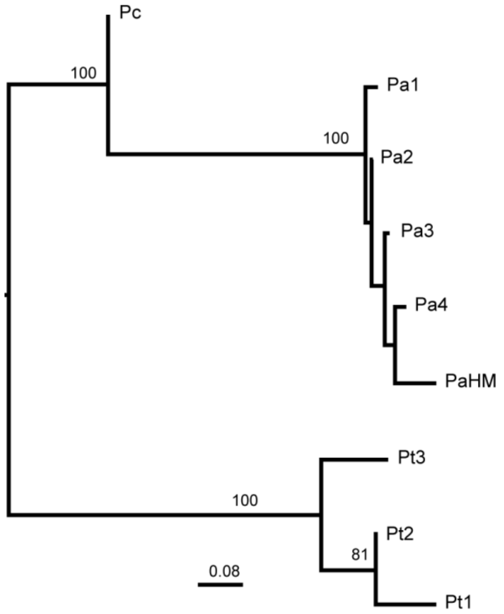

Figure S1. Neighbor-joining tree based on Nei's (1987) distance for microsatellites, including bootstrap support values. Pa: pure *P. alba* populations; Pt: pure *P. tremula* populations; PaHM: *P. alba* coexisting with *P.* × *canescens*; Pc: *P.* × *canescens* population.

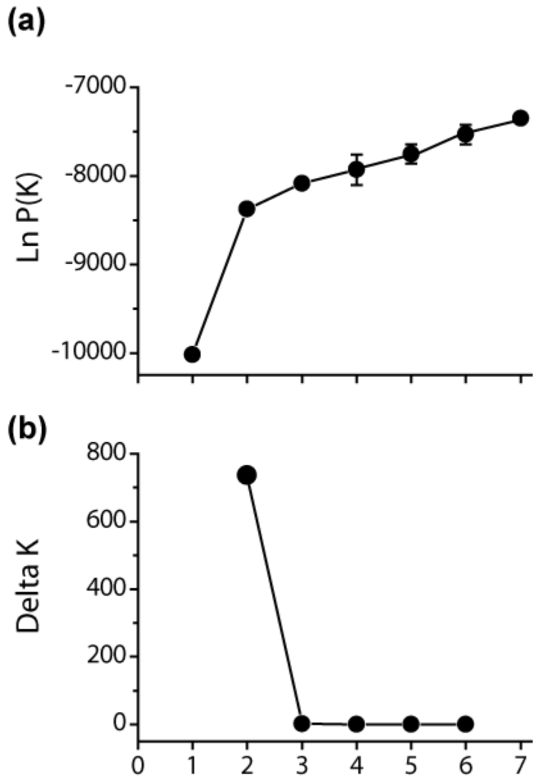

Figure S2. Estimated logarithmic probability (a) of the STRUCTURE analysis and magnitude of ΔK (c) as a function of K for *Populus* individuals from the Irtysh River hybrid zone. Results are from 10 replicates for each of $1 \leq K \leq 7$.

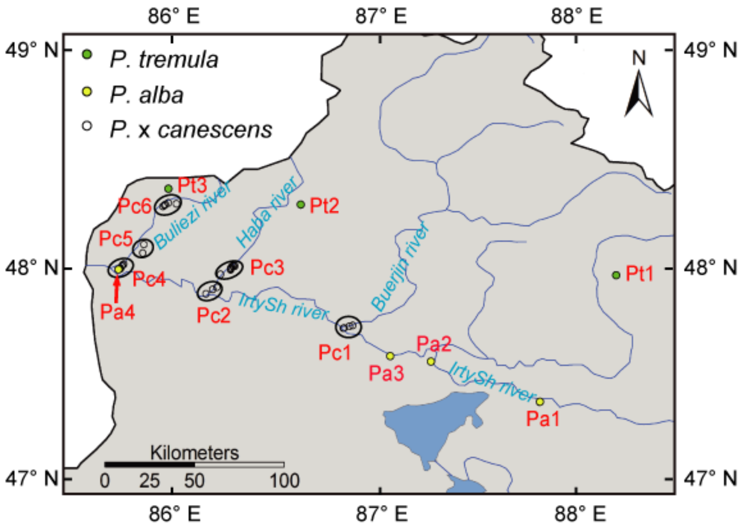

Figure S3. Locations of *Populus* sites sampled along the Irtysh River system. The larger black ovals show the grouping of nearby *P.* × *canescens* sampling sites. The map was created using the ArcMap package in ArcGIS ver. 9.2 (http://www.esri.com/software/arcgis).
